# Effect of Three Interventions on Contact Lens Comfort in Symptomatic Wearers: A Randomized Clinical Trial

**DOI:** 10.1371/journal.pone.0135323

**Published:** 2015-08-12

**Authors:** Maria Navascues-Cornago, Philip B. Morgan, Carole Maldonado-Codina

**Affiliations:** Eurolens Research, Faculty of Life Sciences, The University of Manchester, Manchester, United Kingdom; The Chinese University of Hong Kong, HONG KONG

## Abstract

**Purpose:**

To investigate whether carrying out various interventions part way through the day influences comfort in symptomatic daily disposable (DD) contact lens wearers.

**Methods:**

A subject-masked, randomized, controlled clinical trial was conducted in thirty symptomatic soft lens wearers who wore their habitual DD contact lenses bilaterally for 12 h on two separate days. Five hours after lens application, one of the following three interventions or a control was performed on each eye: replacing the existing lens with a new lens; removing and reapplying the same lens; performing a ‘scleral swish’; and no action (control). Comfort scores were recorded using SMS text messages every hour following lens application using a 0 (causes pain) to 100 (excellent comfort) scale. Comfort scores before lens application, at 6 mins post-application, and at 6 mins post-intervention were also recorded.

**Results:**

There was a significant reduction in comfort from pre-lens application to 6 mins post-application for all groups (all p<0.05). Comfort gradually decreased from 6 mins to 5 h after lens application for each group (p<0.0001) with no significant difference between groups over the 5-h period (p = 0.09). There was no significant difference in comfort 6 mins post-intervention for any group (all p>0.05). After the intervention, comfort continued to decline (p<0.0001) with slightly lower mean scores for the control group compared to the new lens group (p = 0.003). Change in comfort relative to pre-intervention (5 h) was similar for all groups (p = 0.81). There was no difference in comfort at 12 h between groups (p = 0.83).

**Conclusion:**

This work has confirmed that comfort shows a continual and significant decline over a 12-h wearing period in symptomatic DD contact lens wearers. None of the interventions investigated had any significant impact on end-of-day comfort. These data suggest discomfort in lens wearers is more heavily influenced by changes to the ocular environment rather than to the lens itself.

**Trial Registration:**

Controlled-Trials.com ISRCTN10419752 http://www.controlled-trials.com/ISRCTN10419752

## Introduction

The growth of the contact lens industry has been severely limited by the number of discontinuations from lens wear which occur each year. Disappointingly, the number of discontinuations from lens wear approximately equals the number of new fits each year [1]. Contact lens discomfort (CLD) has been consistently reported as the leading cause of lens drop out [2,3] Wearers experiencing CLD usually experience fewer comfortable wearing hours [2,3,4] and they may feel compelled to alter their wearing habits in order to relieve discomfort [5]. This reduced wearing time may lead to temporary periods of lens discontinuation and ultimately to lens drop out [3]. Despite the improvements made by manufacturers over many years in the development of new materials and lens designs as well as the increased availability of daily disposable (DD) contact lenses, comfort during lens wear continues to be problematic, especially towards the end of the wearing period [4,5].

Although extensive research on CLD has been conducted [6,7], the reasons why comfort decreases over the course of the wearing period are not clearly understood. Contact lenses go through changes during wear e.g. dehydration [8,9], surface modification [10,11,12], variation in lens parameters [9,13], and such lens modifications may potentially cause the contact lens to become irritating or uncomfortable. On the other hand, the presence of the contact lens on the eye may cause changes to the ocular tissues that are in direct contact with the lens. Alterations to the ocular surface caused by the lens such as lid parallel conjunctival folds (LIPCOF) [14], lid wiper epitheliopathy (LWE) [15,16], and meibomian gland dysfunction (MGD) [17,18] have been associated with symptoms of discomfort. It is also unclear whether or not CLD is driven by an underlying inflammatory response. The contact lens causes disruption of the tear film which is likely to lead to reduced replenishment of the post-lens tear film in soft contact lens wear [19,20,21]. The stagnation of the post-lens tear film may in turn lead to an increased accumulation of debris, inflammatory cells and other tear film components behind the lens [21,22,23] resulting in increased adverse inflammatory events [24]. Furthermore, some studies have speculated that post-lens debris may induce the release of proinflammatory cytokines [25]. Contact lens wear has been shown to increase the level of certain inflammatory mediators present in the tear film [26,27,28,29,30]. Although there is still little evidence of the influence of inflammatory mediators on CLD, some of these specific tear components such as cytokines may be involved in the generation of pain [31]. Additionally, the interaction between the lens and the lid margin may create friction, which might trigger an inflammatory response. Morgan et al. [32] reported an increase in potential inflammatory cells at the lid margin after contact lens wear which was greater in subjects wearing high-friction contact lenses.

It is still unknown whether end-of-day discomfort is primarily mediated by ocular factors or contact lens factors. If changes to the lens itself trigger symptoms of discomfort, replacing the lens with a new, fresh contact lens should improve the comfort. Recent work by Papas et al. [33] has shown that lens replacement mid-way through a wearing day does not influence end-of-day comfort in hydrogel and silicone hydrogel DD contact lens wearers. The authors have hypothesized that a ‘fatigue-like’ response in the ocular tissues may be induced by the presence of the contact lens. Since symptomatic contact lens wearers differ from asymptomatic wearers in several aspects such as frequency and intensity of symptoms, number of comfortable wearing hours [4], and tear film characteristics [34], this investigation set out to investigate whether such findings also occur in symptomatic lens wearers.

Scleral swish is a procedure performed by some contact lens wearers to replenish the post-lens tear film and to clear debris trapped under the lens. During this procedure the contact lens is slid off of the cornea and onto the temporal conjunctiva. The wearer then blinks a few times and the lens is moved back onto the cornea. Considering that post-lens inflammatory mediators in the tear film could potentially negatively affect comfort, it is of interest to investigate whether performing a scleral swish part way through the wearing day has any impact on end-of-day discomfort.

Subjective scores for lens wearer comfort have traditionally been collected using rating scales at scheduled visits or by subjects annotating their responses in a diary. Devices such as mobile phones present an alternative method of collecting subjective data in that they allow the collection of instant responses and avoid the use of retrospective data [35,36,37,38]. Previous studies have used Short Message Service (SMS) text messages to collect comfort data during contact lens wear [35,37,38]. The use of automated SMS messages allows collection of comfort data at various points throughout the day (e.g. on an hourly basis) and therefore, a more comprehensive monitoring of contact lens comfort throughout a wearing day is possible.

The purpose of this work was to investigate the effect of replacing the lens with the same or with a new lens or performing a scleral swish part way through the wearing day on comfort in symptomatic DD soft contact lens wearers. Assessing the effect of these interventions during the course of a wearing day may allow further insight into what factors (i.e. ocular factors or contact lens factors) mediate end-of-day discomfort.

## Methods

The trial protocol and CONSORT checklist are available as (see S1 Protocol and S1 CONSORT Checklist). Ethical approval for this subject-masked, randomized, controlled clinical trial was obtained from the University of Manchester Committee on the Ethics of Research on Human Beings. The study was conducted at Eurolens Research, The University of Manchester and all subjects received written information about the study before they signed a written statement of consent to participate. The study followed the tenets of the Declaration of Helsinki. This clinical trial was registered retrospectively at ISRCTN10419752. The study was not registered before subject enrolment started since this is not a requirement of our ethics committee. The authors confirm that all ongoing and related trials for this drug/intervention are registered.

### Subjects

As no previous data were available for this work, it was not possible to conduct *a priori* power analysis. Thirty symptomatic DD soft contact lens wearers were recruited and a post-hoc analysis of the power of the work was carried out. Recruitment began in August 2013 and was completed in March 2014. Eurolens Research has a database of subjects who have indicated a willingness to participate in contact lens studies. An export of subject details was filtered according to relevant inclusion/exclusion criteria. Subjects were contacted directly by letter or e-mail. Subjects were also recruited through advertisement on The University of Manchester Research Volunteering website.

### Experimental protocol

#### Screening visit

At a screening visit details of the ocular history were recorded and an anterior eye examination was undertaken to ensure that all subjects met the inclusion and exclusion criteria. Inclusion criteria were of legal age (18 years) and capacity to volunteer, understand their rights as a research subject, *w*illing and able to sign a statement of informed consent, willing and able to follow the protocol, wear daily disposable soft contact lenses and have worn them for a period of at least six months, be classified as symptomatic according by Young et al. method [4], and willing to wear contact lenses for at least 12 hours a day. Exclusion criteria were any systemic or ocular disorder that may affect ocular health, grade 2 or greater of any anterior ocular clinical signs using Efron grading scales [39], use of any topical medication such as eye drops or ointment, previous cataract or corneal refractive surgery, pregnant or lactating and unacceptable contact lens fit (grade -2 or +2 on a -2 to +2 grading scale) [40]. The parameters of subjects’ habitual DD contact lenses were also recorded. Ocular symptoms were assessed using the Contact Lens Dry Eye Questionnaire-8 (CLDEQ-8) [41]. Only subjects who were classified as symptomatic according to the criteria set out by Young et al [4] were recruited. Briefly, subjects are classified as symptomatic based on their responses to the CLDEQ-8 questionnaire on frequency of dryness and intensity of end-of-day dryness. Subjective comfort was assessed using an annotated vertical analogue comfort scale where 0 represented ‘causes pain, cannot be tolerated’ and 100 represented ‘excellent, cannot be felt’. Subjects provided comfort scores for their habitual contact lenses for: comfort at the start and at the end of the day, overall lens comfort and comfort during the study visit. Contact lens wear experience, wearing time of DD lenses, hours per day of lens wear, and days per week of lens wear were recorded. High contrast logMAR visual acuity with contact lenses was measured under high illumination conditions. The following aspects of lens fitting were assessed: horizontal and vertical centration, movement (in the primary gaze position after a blink), and corneal coverage [40]. Deposition, post-lens debris, and lens surface wettability were evaluated using the schema described by Morgan and Efron [42].

#### Study intervention days

Subjects attended the clinic on two further days (there was no wash-out period). On each day, subjects attended the clinic in the morning without any lenses in situ (and not having worn lenses beforehand on the day). A new pair of their habitual DD lenses was applied directly from the packaging solution and worn for at least 12 hours. Comfort scores were recorded before lens application and 6 minutes post-application using the 0 to 100 grading scale. Automated SMS text messages via mobile phone were sent to subjects every hour following lens application requesting a comfort score using the same scale. Five hours after lens application, the investigator performed one of three interventions on each eye together with a control:

1. Replacing the existing lens with a new lens: the existing lens was removed and a new, fresh contact lens was re-applied directly from the packaging solution.
2. Removing the existing lens and reapplying the same lens: the existing lens was removed and placed in a lens case containing 0.9% sterile saline (Eye Care solutions, Crest Medical Ltd, Warrington, UK) for up to one minute to hydrate the lens and then re-applied.
3. Performing a ‘scleral swish’: the contact lens was slid off the cornea onto the temporal conjunctiva. Subjects were then asked to fully blink five times, and then the lens was slid back onto the cornea.
4. No action (control): the contact lens was not manipulated in any way.

The interventions were randomly assigned to each eye (i.e. each eye received a different intervention) and subjects were masked to whether the re-applied lens was a new lens or the same lens. The investigator generated the randomization scheme using the web site Randomization.com (http://www.randomization.com), which created random permutations of interventions for a situation where subjects were to receive all of the interventions in random order.

Comfort scores were recorded 6 minutes post-intervention and subjects continued to respond to hourly SMS messages until they removed their lenses. Comfort scores before lens application, 5 hours post-application, and 6 minutes post-intervention were recorded on paper because subjects were at the clinic at these times. Comfort at 6 minutes post-application at the start of the day was recorded via SMS to confirm that subjects were receiving the text messages on their phones without any problems. Only SMS comfort scores received within 30 minutes from the target time were included in the analysis.

### Statistical analysis

Statistical analysis was carried out using JMP 10 (SAS Institute Inc., Cary, NC). In common with previous literature reports of subjective contact lens comfort scores [33,38,43,44], our comfort data were analyzed using parametric methods. Specifically, a linear mixed model was constructed with the factors of interest being time post-application (Time) and type of intervention (Intervention), subject (as a random effect) and the intervention*time interaction term. Any significant differences were investigated post-hoc with a Student’s t-test. The least square (LS) means are reported. Paired t-test was performed to compare comfort scores before lens application vs. 6 minutes post-application, and pre-intervention vs. 6 minutes post-intervention. The statistical significance level was set at p < 0.05.

## Results

### Subjects

**Fig 1:**
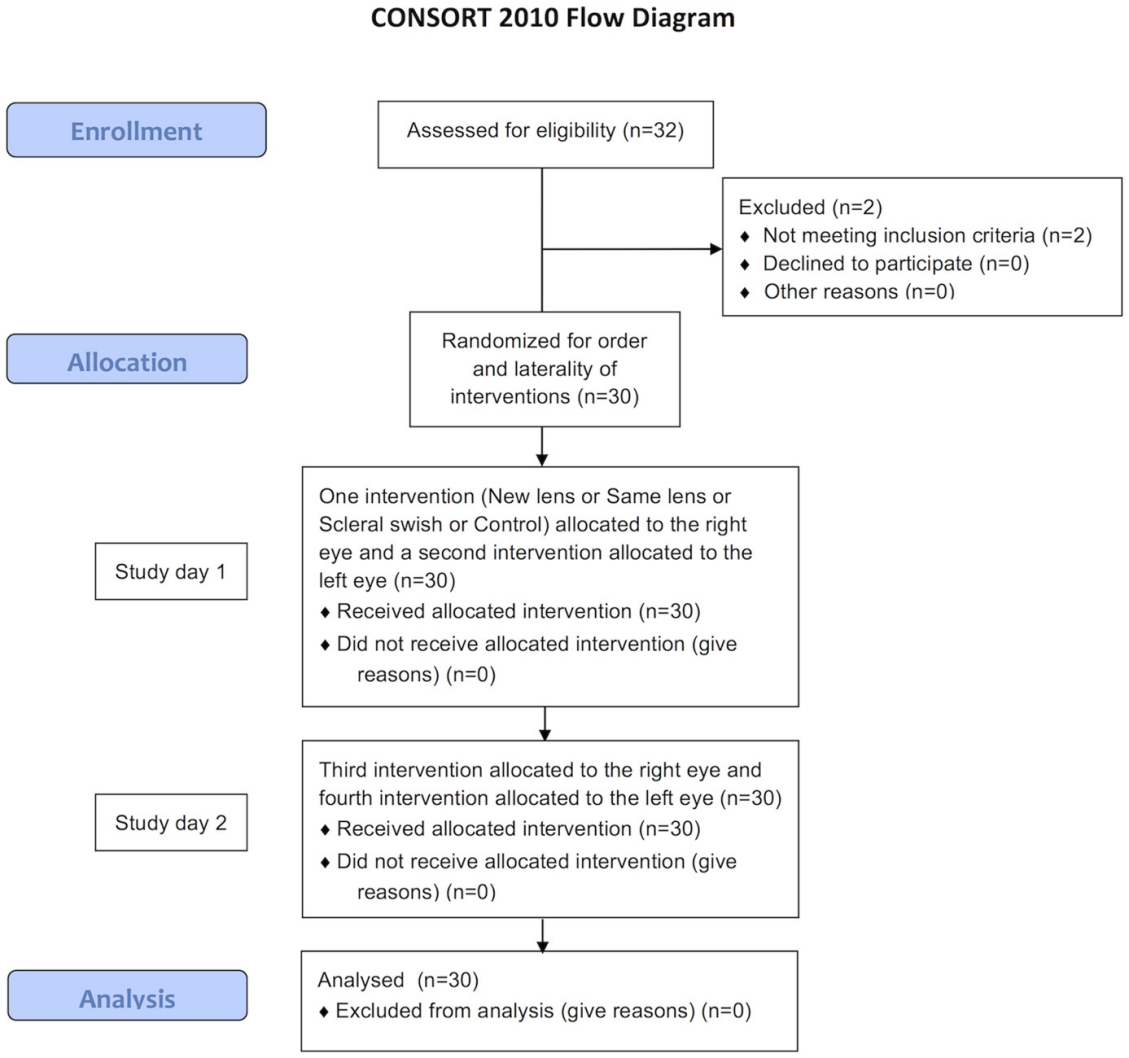
CONSORT Flow Diagram.

Thirty-two subjects were screened for eligibility: two subjects did not meet the inclusion criteria and thirty subjects were recruited and randomly exposed to all the interventions investigated between August 2013 and March 2014 (Fig 1). The subject demographics are shown in Table 1 and their habitual lens parameters are summarized in Table 2.

**Table 1:** Subject demographics (mean ± SD) (range).

Age (years)	28.8 ± 10.9 (18–59)
Gender	22 Female 8 Male
Contact lens experience (years)	7.9 ± 5.3 (1–23)
Daily disposable contact lens experience (years)	6.2 ± 3.6 (1–15)
Days of lens wear per week	4.7 ± 1.4 (2–7)
Hours of lens wear per day	11.3 ± 2.1 (8–16)
Comfortable hours per day	6.9 ± 2.4 (1–12)
CLDEQ-8 score	19.2 ± 4.5 (12–27)
Lens material	23 Hydrogel
	7 Silicone Hydrogel
Lens design	25 Spherical
	3 Toric
	2 Multifocal

**Table 2:** Lens parameters (mean ± SD) (range).

	OD	OS
BOZR (mm)	8.6 ± 0.1 (8.5 to 9)	8.6 ± 0.1 (8.5 to 9)
Total diameter (mm)	14.1 ± 0.2 (13.8 to 14.5)	14.1 ± 0.2 (13.8 to 14.5)
Sphere (D)	-3.03 ± 2.59 (-10 to +3)	-2.87 ± 2.69 (-9.5 to +4.25)
Cylinder (D)	-0.09 ± 0.29 (-1.25 to 0)	-0.08 ± 0.23 (-0.75 to 0)

### Post-hoc power analysis

**Fig 2:**
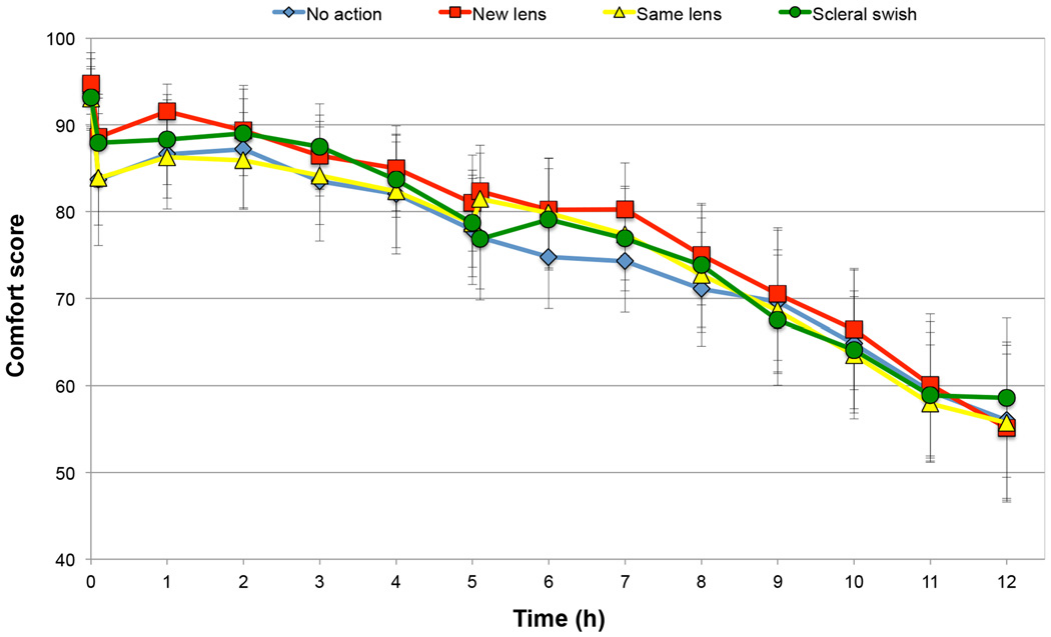
Mean comfort scores over the 12-hour wearing period for all intervention groups. Error bars represent 95% confidence interval.

As no previous data were available for this work, it was not possible to conduct a priori power analysis. However, it is possible to determine the power of the work post hoc. A typical standard deviation of the differences between the no action and new lens interventions was 14.0 units. Assuming a two tailed analysis and an alpha of 0.05, this study had 0.97 power to detect a difference of 10 units; such a difference is considered meaningful given the change in comfort scores presented in Fig 2.

### Screening visit

High contrast logMAR visual acuity with contact lenses was 0.01 ± 0.09 (-0.2 to +0.2). The proportion of optimal fitting characteristics (i.e. grade 0 in all fitting characteristics) was 42% and 58% of lenses showed slightly inadequate to optimal fitting characteristics (i.e. grade -1, 0 or +1 in the fitting characteristics). Most of the lenses showed no surface deposition with only 15% of lenses showing grade 1 (spots). No debris was present in any of the lenses. The wettability was grade 0 (i.e. entirely wettable anterior surface) for 68% of lenses and the remaining lenses were grade 1 (i.e. non-wetting areas of less than 0.1 mm in diameter).

### Study intervention days

**Fig 3:**
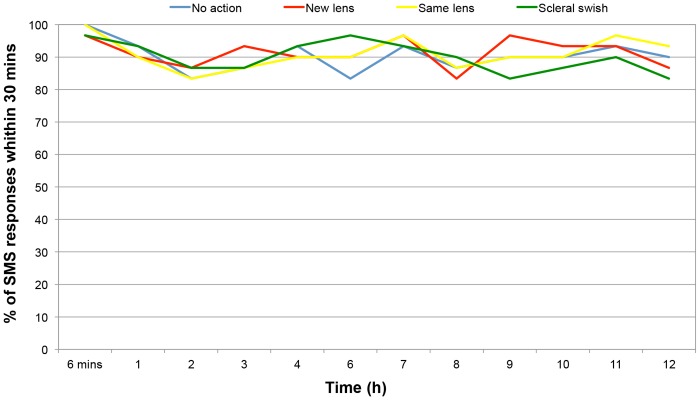
Percentage of SMS responses received within 30 minutes for all intervention groups.

Ninety percent of SMS responses where received within 30 minutes from the target time and the mean ± SD response time was 4.9 ± 6.4 minutes. The percentage of SMS responses received within the acceptable period for each intervention and each time point is shown in Fig 3.

### Comfort scores before the intervention

Comfort scores reported over the 12-hour wearing period for all intervention groups are shown in Fig 2. There was a significant reduction in comfort from pre-lens application to 6 minutes post-application for all intervention groups (paired t-test, all p < 0.05). Table 3 shows the change in comfort (LS mean) at 6 minutes post-application which was similar for all intervention groups (F = 0.9, p = 0.43). There was a significant effect of Time on comfort scores between 6 minutes and 5 hours post-application (F = 39.4, p < 0.0001), but the Intervention and the Intervention*Time interaction were not significant (F = 2.1, p = 0.09; F = 0.2, p = 0.88 respectively).

**Table 3:** Change in comfort (LS mean ± 95% CI) at various intervals of time for all intervention groups.

Time interval	No action	New lens	Same lens	Scleral swish	*P*-value
Pre-lens application to 6 min post-application	-9.9 ± 5.7	-6.5 ± 5.8	-9.2 ± 5.7	-5.5 ± 5.8	0.43
6 min to 5 h post-application	-5.8 ± 7.3	-6.7 ± 7.4	-5.2 ± 7.3	-8.4 ± 7.4	0.26
Pre-intervention (5 h) to 6 min post-intervention	-0.9 ± 5.2	1.3 ± 5.2	2.8 ± 5.2	-1.8 ± 5.2	0.53
Pre-intervention (5 h) to 12 h post-intervention	-22.9 ± 7.4	-26.2 ± 7.6	-23.6 ± 7.3	-20.3 ± 7.7	0.60
6 min to 12 h post-application	-27.9 ± 11.2	-30.5 ± 11.4	-27.6 ± 11.1	-27.2 ± 11.6	0.93

### Comfort scores after the intervention

There was no significant difference in comfort from pre-intervention (i.e. 5 hours) to 6 minutes post-intervention for any group (paired t-test, all p > 0.05). The change in comfort (LS mean) at 6 minutes post-intervention was similar for all the intervention groups (F = 0.7, p = 0.53) (Table 3). Following the intervention, Time and Intervention had a significant effect on comfort scores between 6 minutes post-intervention and 12 hours post-application (F = 353.6, p < 0.0001; F 2.9, p = 0.03 respectively), but the Intervention*Time interaction did not have a significant effect (F = 1.6, p = 0.18). Comfort gradually decreased over this period of time with slightly lower scores (LS mean ± 95% CI) for the no action group compared to the new lens group (68.4 ± 4.9 vs. 72.0 ± 4.9 respectively; Post-hoc p = 0.003).

### Change in comfort relative to pre-intervention

**Fig 4:**
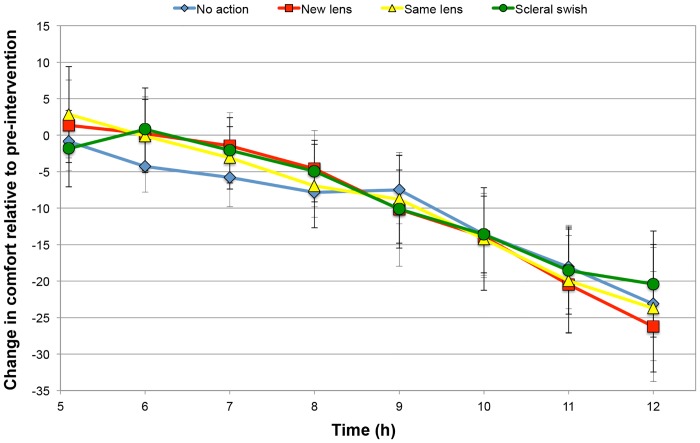
Mean change in comfort relative to pre-intervention (i.e. 5 hours). Error bars represent 95% confidence interval.

Change in comfort relative to pre-intervention (i.e. 5 hours) until the 12-hour time point was also investigated (Fig 4). There was again a significant effect of Time (F = 323.9, p < 0.0001) but the effect of the Intervention and the Intervention*Time interaction were not significant (F = 0.3, p = 0.81; F = 1.6, p = 0.18 respectively). The change in comfort (LS mean) relative to pre-intervention at 12 hours post-application is shown in Table 3.

### Total change in comfort over the 12-hour wearing period

No significant difference in either absolute or relative scores from 6 minutes post-application was found between the intervention groups at 12 hours post-application (F = 0.3, p = 0.83; F = 0.2, p = 0.93 respectively). Table 3 shows the total change in comfort scores over the course of the wearing day (i.e. from 6 minutes to 12 hours post-application).

## Discussion

Many contact lens wearers experience ocular discomfort during lens wear, which is usually more pronounced towards the end of the day [4,5]. The factors that drive this diurnal decrease in comfort are yet to be fully understood. This study reports the effect of replacing the lens with the same or with a new lens or performing a scleral swish part way through the wearing day on comfort in symptomatic DD soft contact lens wearers. The results indicate that the interventions performed had no meaningful impact on end-of-day comfort, which suggest that the decrease in comfort observed in symptomatic DD lens wearers may be driven by ocular factors rather than by lens-related factors.

It is well known that symptomatic contact lens wearers have a more pronounced decline in comfort and increase in dryness symptoms towards the end of the wearing period compared with asymptomatic subjects [8,43,44]. In the present work, the decrease in comfort over a continuous 12-hour wearing period (i.e. no action performed) was approximately 28 units. Differences in the methods used to assess contact lens comfort as well as in the criteria used to classify subjects (i.e. as symptomatic or not) should be born in mind when comparing our results with previous research. However, the reduction in comfort over the day we observed is in agreement with previous studies where symptomatic wearers showed a marked reduction in comfort using visual analogue scales [8,44]. In a study conducted by Fonn et al [8], the reduction in comfort in symptomatic subjects over a 7-hour wearing period was approximately 22 units. Additionally, in a later study by the same author [44], the decrease in comfort in symptomatic lens wearers over a 7-hour wearing period was between 20 and 40 units depending on the lens type. We observed significant variability in our comfort data which is consistent with previous work which have also shown greater variability of comfort ratings in symptomatic lens wearers than in asymptomatic wearers [11,44].

Replacing the lens or performing a scleral swish part way through the wearing day had no clinically significant impact on end-of-day comfort. There was a statistically significant difference in comfort score following the intervention between the new lens group and the control group, with comfort being greater for the new lens group. However, we consider that this small difference in comfort (3.6 units) is clinically insignificant, since it is lower than the difference of 7–8 units (on a 100-point scale) previously reported as the minimum noticeable difference in ocular comfort that contact lens wearers could detect between the two eyes [45]. Similar results have been reported by Papas et al [33], who reported that end-of-day comfort was not influenced by lens replacement in subjects wearing hydrogel and silicone hydrogel lenses. Papas et al [33] reported a marked, although not significant, comfort increase immediately following lens replacement that subsequently dissipated toward the end of day in hydrogel lens wearers. In the present study, none of the interventions investigated had any significant effect on comfort either immediately (i.e. 6 minutes post-intervention) or at the end of the wearing period. Papas et al. [33] assessed comfort at 2–3 minutes after lens replacement, while in the present study comfort was assessed 6 minutes after the intervention had been performed. In addition, the work conducted by Papas consisted of two separate studies: the first study in hydrogel lens wearers (adapted and neophytes) and the second one in silicone hydrogel lens wearers (adapted). In the first study, half of the hydrogel lens wearers replaced their lenses with the same lens and the other half with new lenses, while in the second study all silicone hydrogel wearers were exposed to both scenarios. It is not mentioned in the study whether subjects were symptomatic or asymptomatic. In our study, all subjects were symptomatic adapted lens wearers wearing either hydrogel or silicone hydrogel DD lenses. All subjects were exposed to all the interventions in a contralateral study design. Differences in methodology as well as in subject demographics may explain disparities between studies.

Comfort gradually decreased over the 12-hour wearing period in a similar fashion in all groups regardless of the type of intervention that had been performed. Reapplying the 5-hour worn lens had the same effect on comfort as applying a new, fresh lens, suggesting that changes to the lens during the early part of lens wear (such as dehydration,[8,9] changes to the lens surface [10,11,12], changes to lens parameter [9,13], etc.) do not have a major impact on comfort in DD symptomatic contact lens wearers. Performing a scleral swish, which replenishes the post-lens tear film (and also potentially removes debris and inflammatory mediators from the post-lens tear film) had no significant impact on comfort either. Since this study did not measure inflammatory mediators or post-lens debris directly, it would be erroneous to assume that these factors do not affect contact lens comfort in DD contact lens wearers. Since no data were collected, it can only be hypothesized that the degree of debris or inflammatory mediators developed over short-term wear of DD lenses may not be sufficient to cause the diurnal decrease in contact lens comfort [8,43,44]. Furthermore, the role of inflammation in CLD is still unclear and the fact that adverse symptoms are relieved immediately following removal of the contact lens [5] suggests that other factors may be involved in end-of-day discomfort.

Subjects experienced a steady decrease in comfort which becomes more pronounced at the end of the wearing period (i.e. between 8–9 hours and 12 hours), as indicated by the increased slope of the decrease in comfort being steeper at the end of the wearing period. This pronounced decline was observed in all groups, including the control group (i.e. no action performed), which shows that changes in comfort may be more critical during the last few hours of lens wear. This finding also lends weight to the hypothesis that the contact lens itself induces a ‘fatigue-like’ response in the ocular tissues which was put forward by Papas [33]. Some alterations in the ocular environment related to the presence of a contact lens on-eye that have been associated with CLD include: alterations in the bulbar conjunctiva such as LIPCOF [14]; alterations in the palpebral conjunctiva such as LWE [15,16], and MGD [17,18]; and alteration in the neuroreceptors of the ocular surface [46]. The lid margin has recently received special attention given its interaction with the contact lens surface and its potential important role in contact lens comfort. Future research should be conducted in order to ascertain which ocular tissue is primarily responsible for CLD.

In the present study subjects wore their habitual DD contact lenses, and therefore the blister-pack solution (BPS) could have influenced the results. Packaging solutions incorporate a variety of components such as surfactants and wetting agents in order to improve lens wettability, prevent the lens from sticking to the blister pack and improve initial (or long-term) on-eye comfort [47]. Although the direct effect of the BPS on contact lens comfort has not been investigated in this work, differences in the composition of the BPS between the different lenses [47] could in theory have affected (either positively or negatively) comfort results for the intervention where the existing lens was replaced by a new lens which was applied directly from the blister packaging.

A contralateral design was used in the present study. The fact that different interventions were performed in different eyes of the same subject may have induced a confounding effect on the comfort responses. Previous studies have shown that there is a physiological contralateral effect, in that the intervention performed in one eye may have an impact on the other eye [48]. However, we believe that this contralateral effect is mitigated to a large extent by the randomization employed in the study design. Additionally, simultaneous exposure of both eyes to different interventions might allow a more direct comparison between interventions.

The use of electronic devices such as mobile phones has become an alternative method of collecting subjective data in clinical trials [35,36,37,38]. The use of SMS text messages allows collection of contact lens comfort in real time and avoids the use of retrospective data. In this work using hourly text messages, 90% of messages were received within 30 minutes of the scheduled time point and the mean time response was 5 minutes. These results are consistent with those reported by previous studies where SMS messaging was used [35,37]. The high response rate and the rapid response confirm that the use of SMS via mobile phone is a very successful and efficient method of collecting subjective data that provides a comprehensive understanding of the changes in contact lens comfort over the course of an entire wearing day.

In summary, comfort showed a continual and significant decline over a 12-hour wearing period in the symptomatic DD contact lens wearers who took part in this work. None of the interventions investigated had any clinically significant impact on end-of-day comfort, which suggests that discomfort in lens wearers may be more heavily influenced by changes to the ocular environment rather than to the lens itself. Future research should be directed towards understanding which ocular tissue(s) is primarily responsible for driving the reduction in comfort typically over the course of a contact lens wearing day.

## Supporting Information

S1 Protocol Trial Protocol. (PDF)

S1 CONSORT Checklist CONSORT Checklist. (DOCX)
