# Supplementary material for: Effect of Three Interventions on Contact Lens Comfort in Symptomatic Wearers: A Randomized Clinical Trial
Source: PLoS One. 2015 Aug 12;10(8):e0135323. doi: 10.1371/journal.pone.0135323 (PMC4533967; doi:10.1371/journal.pone.0135323)
Supplement: S1 Protocol — (PDF) [file pone.0135323.s001.pdf]

Clinical investigation plan
ER13-541

Effect of lens replacement and manipulation on comfort in contact lens wear

Principal Investigators
Philip B. Morgan
Carole Maldonado-Codina

March 2013

Contents

**Section 1.**	**Overview**	**5**
	1.1 Background	5
	1.2 Personnel	5
	1.3 Study objectives	6
	1.4 Study design	6
	1.5 Statistical considerations	6
	1.6 Risk analysis	6
**Section 2.**	**Resources**	**7**
	2.1 Subject selection	7
	2.2 Subject discontinuation	8
	2.3 Safety parameters, adverse events and concurrent illnesses	8
	2.4 Study termination	9
	2.5 Protocol deviations	9
	2.6 Study resources	9
	2.7 Study control	9
	2.8 Documentation	10
	2.9 Data collection and analysis	10
	2.10 Study completion	10
	2.11 Confidentiality	10
**Section 3.**	**Subject management**	**11**
	3.1 Visit scheduling	11
	3.2 Visit conduct	11
	3.3 Monitoring subject compliance	14
	3.4 Missing, unused and spurious data	14
**Section 4.**	**Study co-ordination**	**15**
	4.1 Personnel	15
**Section 5.**	**References**	**18**

Appendices

A Adverse events
B Randomisation tables
C Information and consent forms, grading scales
D Recruitment

Document control

Study title:	Effect of lens replacement and manipulation on comfort in contact lens wear (ER13-541)
Sponsor:	The University of Manchester
Funding body:	The University of Manchester
Document type:	Clinical Investigation Plan

Document approved by: Carole Maldonado-Codina (signature) Date March 8, 2013

Carole Maldonado-Codina
Principal Investigator

Document reviewed and approved by: P. Morgan (signature) Date March 8, 2013

Philip B. Morgan
Principal Investigator

Study summary

The purpose of this work is to investigate the effect of various interventions i.e. lens replacement (during a lens wearing day) and lens manipulation on comfort in contact lens wear.

Up to 30 soft contact lens wearers will be recruited for this study. All volunteers will be symptomatic (i.e. classified as symptomatic according to method of Young et al.[(1)]). Subjects will attend the clinic for six visits over four days. Subjects will wear their habitual contact lenses during the course of this investigation. Various interventions i.e. lens replacement and lens manipulation [i.e. the contact lens is slid off the cornea onto the temporal sclera and then repositioned (this is known as the 'scleral swish')] will be performed in order to assess their impact on lens discomfort. Comfort scores will be collected at different times of the day mainly via SMS (text) messaging.

A study summary is shown in Table 1.

Visit		Procedures
Day 1	Visit 1	Explanation of study procedures and visits<br>Confirmation that all inclusion/exclusion criteria are met<br>Information and consent forms and study summary signed<br>Ocular history<br>Ocular symptoms measurement<br>Visual acuity measurement<br>Lens fit assessment<br>Slit lamp biomicroscopy
Day 2	Visit 2 (morning)	Comfort score prior to lens application both eyes<br>Lens application<br>Comfort score after 6 minutes both eyes
	Visit 3 (5 hours later)	Lens replacement or lens manipulation on each eye<br>Comfort scores after 6 minutes both eyes
Day 3	Visit 4 (morning)	Comfort score prior to lens application both eyes<br>Lens application<br>Comfort score after 6 minutes both eyes
	Visit 5 (5 hours later)	Lens replacement or lens manipulation on each eye<br>Comfort scores after 6 minutes both eyes
Day 4	Visit 6	Visual acuity measurement<br>Slit lamp biomicroscopy<br>Exit form completed

Table 1: Study summary.

Section 1. Overview

1.1 Background

Discomfort with soft contact lenses remains a major issue for the industry,[2, 3] giving rise to millions of discontinuing wearers each year. Indeed, the key reason to discontinuation from lens wear is some form of discomfort.[4, 5] Despite the obvious commercial benefits of better knowledge in this area, a comprehensive understanding of the determinants of contact lens comfort remains elusive. Over the past 20 years, various investigators have sought to relate factors such as lens design (e.g. nature of the lens edge,[6, 7] dehydration,[8, 9] and tear film thickness/stability[10, 11] to contact lens comfort; however, such factors do not appear to fully explain the nature of contact lens comfort.

Frequency of lens replacement may influence comfort in contact lens wear.[12-15] Several studies have shown that when contact lenses are replaced more frequently, comfort ratings increase.[12-15] Symptoms of discomfort in contact lens wear may increase towards the end of the day.[1, 16, 17] Assessing the effect of replacing contact lenses during a lens wearing day may allow further insight into what factors (i.e. ocular factors or contact lens factors) mediate end of day discomfort.

The purpose of this work is to investigate the effect of various interventions i.e. lens replacement and manipulation during a lens wearing day on the comfort of a group of symptomatic contact lens wearers. Subjects will wear their habitual contact lenses during the course of the study. Various interventions i.e. lens replacement and lens manipulation ('scleral swish') will be performed in order to assess their impact on lens discomfort. On two separate days four interventions (chosen randomly) will be performed. All volunteers will be symptomatic (i.e. classified as symptomatic according to method of Young et al.[1]). We will collect contact lens comfort scores at different times of the day mainly via SMS (text) messaging.

1.2 Personnel

This work will be conducted at Eurolens Research, The University of Manchester under the general direction of Philip Morgan PhD MCOptom FAAO FBCLA. The Principal Investigators for the work are Philip Morgan and Carole Maldonado-Codina PhD MCOptom FAAO FBCLA. Subjects will be examined by Maria Navascues-Cornago who is an optometrist and a PhD student at The University of Manchester. This project will be supervised by Philip Morgan and Carole Maldonado-Codina. Clinical work carried out by

the student will be supervised by a GOC registered optometrist and this will usually be Philip Morgan or Carole Maldonado-Codina. The following optometrists may also be involved in supervising this practical work and examining subjects: Neil Chatterjee MSc MCOptom, Gillian Howarth BSc(Hons) MCOptom, Aftab Mirza MSc MCOptom and Michael Read PhD MCOptom. Project management and data analysis will be the responsibility of Maria Navascues Cornago with input from the two supervisors. Data management support will be provided by Andrew Plowright. Further support such as administration and support with recruitment of subjects will be provided by Tonie Sly, Kate Wickstone, Theresa Rigg, Tracey Burgoyne and Francesca Pritchard.

1.3 Study objectives

This study seeks to investigate the effect of various interventions i.e. lens replacement and manipulation during a lens wearing day on comfort in contact lens wear.

1.4 Study design

This clinical study will be a randomised and subject-masked (interventions and comfort scores) study which will take place at a single study site. Up to 30 soft contact lens wearers will be recruited. Subjects will wear their habitual contact lenses during the course of this investigation. Various interventions i.e. lens replacement and manipulation will be performed in order to assess their impact on lens discomfort. Comfort scores will be collected at different times of the day mainly via SMS (text) messaging.

1.5 Statistical considerations

The principal hypotheses to be tested in this work is that ocular comfort scores will be substantially equivalent for all the interventions assessed.

Given the lack of previous data with which to conduct a priori calculations for subject numbers for the study, we decided to use up to 30 subjects as we think this number of subjects is sufficient to for the project to be worthwhile.

1.6 Risk analysis

The risks in this study are considered to be similar to those of normal contact lens wear. With the potential benefit of this study, the work is considered to be ethically justifiable. Ethical approval will be sought from The University of Manchester Senate Committee on the Ethics of Research on Human Beings (hereafter referred to as Manchester UREC). The work where practical will be conducted in accordance with the ICH Good Clinical Practice Guidelines and the international standard BS EN ISO 14155:2011 ‘Clinical investigation of medical devices for human subjects’.

Section 2. Resources

2.1 Subject selection

In this work up to 30 soft contact lens wearers will be recruited.

2.1.1 Subject withdrawal and replacement

Once the study consent form is signed, the subject is considered to be enrolled on the study. Subjects who have signed the consent form, but who have not completed the first visit will usually be replaced. All subject data will be included in the final analyses unless there are strong grounds for exclusion; such grounds will be detailed in the final report.

2.1.2 Subject recruitment

Subjects will be recruited by one or more of following means:

1. Correspondence to existing wearers on the Eurolens Research database of subjects.
2. Advertising through a variety of media via a format separately approved by Manchester UREC.

Samples of correspondence can be found in Appendix D.

2.1.3 Inclusion criteria

Subjects will only be eligible for the study if:

1. They are 18 years of age and above.
2. They understand their rights as a research subject and are willing and able to sign a Statement of Informed Consent.
3. They are willing and able to follow the protocol.
4. They agree not to participate in other clinical research for the duration of this study.
5. They currently use daily disposable soft contact lenses and have used them for a period of at least six months.
6. They are classified as symptomatic according to method of Young et al. [(1)].
7. They are willing to wear their contact lenses for at least 12 hours a day.
8. They have a mobile phone

2.1.4 Exclusion criteria

Subjects will not be eligible to take part in the study if:

1. They have an ocular disorder.
2. They have a systemic disorder which may affect ocular health.

3. They are using any topical medication such as eye drops or ointment.
4. They have had cataract surgery.
5. They have had corneal refractive surgery.
6. They have any corneal distortion resulting from previous hard or rigid lens wear or have keratoconus.
7. They are pregnant or lactating.
8. They have diabetes.
9. They have grade 2 or greater of any of the following ocular surface signs: corneal oedema, corneal vascularisation, corneal staining, tarsal conjunctival changes or any other abnormality and which would normally contraindicate contact lens wear.
10. They have any infectious disease (e.g. hepatitis) or any immunosuppressive disease (e.g. HIV) or a history of severe allergic reaction or anaphylaxis (a serious form of hypersensitivity which can, in rare cases, result in death).
11. They have worn soft contact lenses for less than six months.
12. The lens fit is unacceptable (grade -2 or +2 on a -2 to +2 grading scale (Appendix C).
13. They have taken part in any other clinical trial or research, within two weeks prior to starting this study.

2.2 Subject discontinuation

In general, subjects should be discontinued at any time, if it is in their best interests, as judged by the investigator. Reasons for this may include clinical signs of grade 3 or more, lack of motivation, discomfort or repeated refusal to follow instructions or the use of contact lenses during the study period. Subjects will be discontinued if a serious adverse event occurs. Subjects who fail to satisfy all the inclusion and exclusion criteria will be discontinued and replaced. Subjects may choose to leave the study at their own request. All discontinuations will be carefully recorded.

2.3 Safety parameters, adverse events and concurrent illnesses

The key safety parameters are the serious and significant adverse events listed in Appendix A (adverse events are classified as ‘serious’, ‘significant’ or ‘non-significant’). Clinical assessment is made at the study visit(s) for these parameters. The presence of any adverse event will be reported on the case report forms and those described as ‘serious’ or ‘significant’ will be detailed in the final report. Similarly, any concurrent illness that is likely to impact on the relevance and quality of the captured data will be noted on the case report form.

2.3.1 Investigator obligations

At all times the investigator will act in the best interest of the subject. Referral or treatment of an adverse event or other clinical finding should be initiated in the best clinical judgement of the investigator, irrespective of the participation in the clinical study.

2.3.2 Reporting obligations

In the case of a ‘serious’ or ‘significant’ adverse event, Manchester UREC and any regulatory authorities will be informed as required.

2.4 Study termination

If it becomes necessary to terminate the study earlier than planned, Manchester UREC will be informed.

2.5 Protocol deviations

Any deviations from this protocol will be recorded and reported to Manchester UREC.

2.5.1 Protocol amendments

Any amendments will be recorded, identified and distributed. Approval from Manchester UREC will be obtained.

2.6 Study resources

Subjects will not be issued with study products in this investigation.

2.6.1 Lenses

Subjects will use their own daily disposable contact lenses. A description of the various interventions to be investigated in this study are shown in Table 2.

Interventions	Description
Intervention 1	No intervention
Intervention 2	The contact lens will be removed and replaced by a fresh contact lens.
Intervention 3	The contact lens will be removed and replaced by the same contact lens.
Intervention 4	The contact lens will be slid off the cornea onto the temporal sclera and then repositioned (‘scleral swish’)

Table 2: Description of interventions

2.6.2 Clinical equipment

Clinical equipment is regularly maintained and calibrated as required. Standard operating procedures and international standards are used where appropriate.

2.7 Study control

Bias will be minimised by randomising the order of interventions.

2.8 Documentation

Documents related to this work that require archiving will be kept by Eurolens Research for a period of 10 years after completion of the final report, before being destroyed.

2.9 Data collection and analysis

Comfort scores will be collected mainly via SMS (text) messaging. Data collected in this work will be recorded on paper case report forms (source data). Data handling will include the input of the study information from the case report forms into spread sheet format for manipulation, followed by export into a statistical package for analysis.

2.10 Study completion

The study is complete when all subjects have signed the exit statement.

2.11 Confidentiality

All matters related to this work will remain confidential within Eurolens Research and any regulatory authority (e.g. Manchester UREC). Eurolens Research will take all reasonable steps to ensure that specific information is not passed on to study participants unless this is required for clinical management of an adverse event. Personal subject information will not be made available. To cater for this, subjects will only be referred by their unique identity number in the study report. The data activities of Eurolens Research are registered with the data protection officer at The University of Manchester.

Section 3. Subject management

3.1 Visit scheduling

Up to 30 subjects will take part in this study. Subjects will attend the clinic for six visits over four separate days: initial visit on Day 1; Day 2 (in the morning and five hours later); Day 3 (in the morning and five hours later); and exit visit on Day 4. The maximum period between Day 1 and Day 4 will be three weeks.

3.1.1 Unscheduled visits

Subjects who attend a visit outside of the allowable range as described above will be classed as having had an 'unscheduled visit'. Additionally, subjects who attend at their own volition, (or as instructed to do so by the investigator) rather than for a scheduled study visit, will be examined and the visit will be classified as 'unscheduled'.

3.1.2 Missed visits

Subjects not attending for a visit will be contacted and encouraged to return for assessment.

3.2 Visit conduct

3.2.1.1 Day 1: visit 1.

The subject will receive a study-specific information form outlining the study at least 24 hours before the initial visit, and will be required to sign an informed consent form and a study summary form at this visit, prior to enrolment (Appendix C). Copies of the signed forms will be issued to the subject. When the subject has signed the consent form, they are considered to be enrolled on the study. Subjects should be asked to attend this visit with their lenses in situ and to bring their habitual care regimen with them.

The following procedures will be performed:

1. Details of the ocular history.
2. The investigator will confirm that the subject satisfies all the inclusion and exclusion criteria. Subjects who fail to meet all the criteria at this time will be discontinued and replaced.
3. High contrast logMAR visual acuity in accordance with the current Eurolens Research Standard Operating Procedure 'Assessment of visual performance using the Bailey-Lovie logMAR visual acuity test chart and procedures for carrying out an over-refraction'.

4. Ocular symptoms will be measured using Contact Lens Dry Eye Questionnaire - 8 (CLDEQ-8) and 100-point vertical visual analogue scales (Appendix C).
5. Lens fit will be assessed using the following evaluations: horizontal and vertical centration, corneal coverage and movement. Normally, for an acceptable fit, centration and movement will fall within currently accepted clinical criteria [between -1 and +1 on a -2 to +2 grading scale (Appendix C)]. Lens deposition and wettability will also be graded using the grading scales in Appendix C.
6. Slit lamp biomicroscopy will be carried out for the signs outlined in Table 2 and in accordance with the current Eurolens Research Standard Operating Procedure 'Examination of the anterior segment using slit lamp biomicroscopy'. Grades will be scored to the nearest 0.1 in the best judgement of the investigator using Efron Grading Scales.

Classification	Primary signs	Secondary signs
Signs	Conjunctival redness<br>Limbal redness<br>Corneal neovascularisation<br>Epithelial microcysts<br>Corneal oedema<br>Corneal staining<br>Location of staining<br>Conjunctival staining<br>Papillary conjunctivitis	Blepharitis<br>Meibomian gland dysfunction<br>Mucin balls<br>Lid wiper epitheliopathy
Scale	Efron Grading Scales (scored to nearest 0.1)	Efron Grading Scales (scored to nearest 0.1) (except mucin balls, where the number is recorded and lid wiper epitheliopathy, where a dedicated grading scale will be used).

Table 3: Biomicroscopic signs.

7. The presence of any adverse events will be recorded (Appendix A).
8. The subject will be discharged and asked to attend the next scheduled visit.

3.2.1.2 Day 2

3.2.1.2.1 Visit 2

Subjects will attend the clinic in the morning. They will be asked to attend the clinic without lenses in situ, but to bring their lenses with them. Comfort scores will be recorded (on paper) prior to lens application. Contact lenses will be applied and comfort scores will be collected after 6 minutes via SMS. Subjects will wear their contact lenses for at least 12 hours and will provide comfort scores at various times of the day (Table 4) via SMS messaging using the same rating scale. Subjects will be asked to return 5 hours after lens application.

Time of the day	Comfort measurement
Prior to lens application	Paper
6 minutes after lens application	SMS
1 hour after lens application	SMS
2 hours after lens application	SMS
3 hours after lens application	SMS
4 hours after lens application	SMS
5 hours after lens application	Paper
6 min after intervention	Paper
6 hours after lens application	SMS
7 hours after lens application	SMS
8 hours after lens application	SMS
9 hours after lens application	SMS
10 hours after lens application	SMS
11 hours after lens application	SMS
12 hours after lens application	SMS
Every hour until lens removal	SMS

Table 4: Times of comfort measurement

3.2.1.2.2 Visit 3

Subjects will return to the clinic 5 hours after lens application. The investigator will perform the intervention randomly assigned to each eye (Appendix C). The investigator will make every effort to keep the intervention masked from the subject. Comfort scores will be recorded on paper prior to the intervention and 6 minutes after the intervention. Subjects will then be asked to continue wearing their lenses and to provide comfort scores via SMS (Table 4) until they remove their lenses.

Subjects will be asked to attend the next scheduled visit (Study Day 2).

3.2.1.3 Day 3

3.2.1.3.1 Visit 4

The same procedures as at the Study Visit 1 will be carried out.

3.2.1.3.2 Visit 5

The same procedures as at the Study Visit 2 will be carried out but the interventions will differ from that at Study Visit 2 (Appendix B). At the end of the visit, subjects will be asked to attend the next scheduled visit (Exit Visit).

3.2.1.4 Day 4: visit 6.

Subjects will be asked to attend this visit without lenses in situ, but to bring their lenses and habitual care regimen with them.

The following procedures will be performed:

1. Slit lamp biomicroscopy as outlined in 3.2.1.
2. logMAR visual acuity (high contrast) as outlined in 3.2.1.
3. Subjects will then be exited from the study. The subject will sign a study exit statement acknowledging that the work is complete, although they may have been asked by the investigator to attend a post-study follow-up visit, and that they should continue to use their lenses and solutions as advised, and seek aftercare for their contact lenses. A copy of this signed form will be issued to the subject.

3.2.2 Post-study follow-up visit

In the case of a subject who exits the study with significant clinical signs or symptoms, the investigator must undertake to examine the subject at intervals he/she determines to be clinically appropriate until the sign or symptom has resolved or returned to a level that is considered to be clinically acceptable. Details from these visits will be recorded on a post-study follow-up visit form.

3.3 Monitoring subject compliance

Subjects are required to adhere to the instructions provided during this clinical investigation. This will be confirmed at the study visits by verbal questioning of the subject by the investigator.

3.4 Missing, unused and spurious data

The absence of any data will be carefully and critically considered. If appropriate, partial datasets will be included in the final analysis. Any data missing from a subject visit will be outlined in the report by indicating the number of subjects included for each analysis. Data that are unused or considered to be spurious will be detailed and discussed in the report.

Section 4. Study co-ordination

4.1 Personnel

Principal Investigators

Philip Morgan PhD MCOptom FAAO FBCLA
Eurolens Research
Faculty of Life Sciences
Carys Bannister Building
The University of Manchester
Dover St
Manchester
M13 9PT
Telephone +44 161 306 8761 Fax +44 161 200 4442
E-mail philip.morgan@manchester.ac.uk

Carole Maldonado-Codina PhD MCOptom FAAO FBCLA
Eurolens Research
Telephone +44 161 306 4441 Fax +44 161 200 4442
E-mail c.m-codina@manchester.ac.uk

Student Investigator

Maria Navascues-Cornago BSc MSc
E-mail maria.navascuescornago@postgrad.manchester.ac.uk

Eurolens Research Investigators

Neil Chatterjee MSc MCOptom
Eurolens Research
Telephone +44 161 306 4431 Fax +44 161 200 4442
E-mail n.chatterjee@manchester.ac.uk

Gillian Howarth BSc(Hons) MCOptom
Eurolens Research
Telephone +44 161 306 3880 Fax +44 161 200 4442
E-mail gillian.howarth@manchester.ac.uk

Aftab Mirza MSc MCOptom

Eurolens Research
Telephone +44 161 306 4437 Fax +44 161 200 4442
E-mail aftab.mirza@manchester.ac.uk

Michael Read PhD MCOptom
Eurolens Research
Telephone +44 161 306 8762 Fax +44 161 200 4442
E-mail michaelread@manchester.ac.uk

Operations Manager

Andrew Plowright M.Sc.
Eurolens Research
Telephone +44 161 306 8762 Fax +44 161 200 4442
E-mail Andrew.plowright@manchester.ac.uk

Project Officers

Tonie Sly BSc(Hons)
Eurolens Research
Telephone +44 161 306 8762 Fax +44 161 200 4442
E-mail tonie.sly@manchester.ac.uk

Michelle Inwood BSc(Hons)
Eurolens Research
Telephone +44 161 306 4443 Fax +44 161 200 4442
E-mail michelle.inwood@manchester.ac.uk

Research Administrators

Tracey Burgoyne
Eurolens Research
Telephone +44 161 306 3861 Fax 0870 1 123 266
E-mail t.burgoyne@manchester.ac.uk

Theresa Rigg
Eurolens Research
Telephone +44 161 306 2132 Fax +44 161 200 4442
E-mail theresa.rigg@manchester.ac.uk

Kate Wickstone
Eurolens Research
Telephone +44 161 306 2132 Fax +44 161 200 4442
E-mail kate.wickstone@manchester.ac.uk

Francesca Pritchard
Eurolens Research
Telephone +44 161 306 2132 Fax +44 161 200 4442
E-mail Francesca.pritchard@manchester.ac.uk

Section 5. References

1. Young G, Chalmers RL, Napier L, Hunt C, Kern J. Characterizing contact lens-related dryness symptoms in a cross-section of UK soft lens wearers. Cont Lens Anterior Eye. 2011 Apr;34(2):64-70.
2. Begley CG, Chalmers RL, Mitchell GL, Nichols KK, Caffery B, Simpson T, et al. Characterization of ocular surface symptoms from optometric practices in North America. Cornea. 2001;20(6):610-8.
3. Begley CG, Caffery B, Nichols KK, Chalmers R. Responses of contact lens wearers to a dry eye survey. Optometry and Vision Science. 2000;77(1):40-6.
4. Pritchard N, Fonn D, Brazeau D. Discontinuation of contact lens wear: a survey. Int Contact Lens Clin. 1999;26:157-62.
5. Richdale K, Sinnott LT, Skadahl E, Nichols JJ. Frequency of and factors associated with contact lens dissatisfaction and discontinuation. Cornea. 2007 FEB;26(2):168-74.
6. Maïssa C, Guillon M, Garofalo RJ. Contact lens-induced circumlimbal staining in silicone hydrogel contact lenses worn on a daily wear basis. Eye & Contact Lens: Science & Clinical Practice. 2011.
7. Santodomingo-Rubido J, Wolffsohn J, Gilmartin B. Conjunctival epithelial flaps with 18 months of silicone hydrogel contact lens wear. Eye & Contact Lens: Science & Clinical Practice. 2008;34(1):35-8.
8. Ousler GW, Anderson RT, Osborn KE. The effect of senofilcon A contact lenses compared to habitual contact lenses on ocular discomfort during exposure to a controlled adverse environment. Curr Med Res Opin. 2008;24(2):335-41.
9. Pritchard N, Fonn D. Dehydration, lens movement and dryness ratings of hydrogel contact lenses. Ophth Physiol Opt. 1995;15(4):281-6.
10. Geldis JR, Nichols JJ. The impact of punctal occlusion on soft contact lens wearing comfort and the tear film. Eye Contact Lens. 2008;34(5):261-5.
11. Peterson RC, Wolffsohn JS, Nick J, Winterton L, Lally J. Clinical performance of daily disposable soft contact lenses using sustained release technology. Cont Lens Anterior Eye. 2006 Jul;29(3):127-34.
12. Solomon OD, Freeman MI, Boshnick EL, Cannon WM, Dubow BW, Kame RT, et al. A 3-year prospective study of the clinical performance of daily disposable contact lenses compared with frequent replacement and conventional daily wear contact lenses. CLAO J. 1996 Oct;22(4):250-7.
13. Fahmy M, Long B, Giles T, Wang C-H. Comfort-enhanced daily disposable contact lens reduces symptoms among weekly/monthly wear patients. Eye & Contact Lens: Science & Clinical Practice. 2010;36(4):215-9.
14. Malet F, Schnider CM. Influence of replacement schedule and care regimen on patient comfort and satisfaction with daily wear frequent-replacement contact lenses. CLAO J. 2002 Jul;28(3):124-7.
15. Pritchard N, Fonn D, Weed K. Ocular and subjective responses to frequent replacement of daily wear soft contact lenses. CLAO Journal. 1996;22(1):53-9.
16. Santodomingo-Rubido J, Barrado-Navascues E, Rubido-Crespo MJ. Ocular surface comfort during the day assessed by instant reporting in different types of contact and non-contact lens wearers. Eye Contact Lens. 2010 Mar;36(2):96-100.
17. Fonn D, Dumbleton K. Dryness and discomfort with silicone hydrogel contact lenses. Eye Contact Lens. 2003 Jan;29(1 Suppl):S101-4; discussion S15-8, S92-4.
